# Evaluation of the Create@School Game-Based Learning–Teaching Approach

**DOI:** 10.3390/s19153251

**Published:** 2019-07-24

**Authors:** Eugenio Gaeta, María Eugenia Beltrán-Jaunsaras, Gloria Cea, Bernadette Spieler, Andrew Burton, Rebeca Isabel García-Betances, María Fernanda Cabrera-Umpiérrez, David Brown, Helen Boulton, María T. Arredondo Waldmeyer

**Affiliations:** 1 LifeSTech, Department of Photonics and Bioengineering, Escuela Técnica Superior de Ingenieros de Telecomunicación, Universidad Politécnica de Madrid, Avenida Complutense nº 30, Ciudad Universitaria 28040 Madrid, Spain; 2 Graz University of Technology, Institute for Software Technology, Inffeldgasse 13/V, 8010 Graz, Austria; 3 Nottingham Trent University, 50 Shakespeare Street, Nottingham NG1 4FQ, UK

**Keywords:** education, coding Apps, coding, STEM, pocket code, Create@School, mobile sensors, LEGO® sensors

## Abstract

The constructivist approach is interested in creating knowledge through active engagement and encourages students to build their knowledge from their experiences in the world. Learning through digital game making is a constructivist approach that allows students to learn by developing their own games, enhancing problem-solving skills and fostering creativity. In this context two tools, Create@School App and the Project Management Dashboard (PMD), were developed to enable students from different countries to be able to adapt their learning material by programming and designing games for their academic subjects, therefore integrating the game mechanics, dynamics, and aesthetics into the academic curriculum. This paper focuses on presenting the validation context as well as the evaluation of these tools. The Hassenzahl model and AttrakDiff survey were used for measuring users’ experience and satisfaction, and for understanding emotional responses, thus providing information that enables testing of the acceptability and usability of the developed apps. After two years of usage of code-making apps (i.e., Create@School and its pre-design version Pocket Code), the pupils processed knowledge from their academic subjects spontaneously as game-based embedded knowledge. The students demonstrated creativity, a practical approach, and enthusiasm regarding making games focused on academic content that led them to learning, using mobile devices, sensors, images, and contextual information. This approach was widely accepted by students and teachers as part of their everyday class routines.

## 1. Introduction

The constructivist approach [1] is interested in building knowledge through active engagement. This approach suggests that students learn best by making artefacts that can be shared with others. It encourages students to construct their knowledge from their experiences in the real world. The constructivist approach [2] goes a step further and argues that students learn the best not only when they create their own artefacts, but also when they share them with others. Designing sharable artefacts on their own reflects students’ different styles of thinking and learning.

### 1.1. The Learning Theory and Game-Based Learning

Learning through game making is a constructivist approach that allows students to develop their own games [3], enhancing their problem-solving skills and fostering creativity [4]. The learners should be able to develop and adapt their learning material by programming and designing games for their academic subjects, therefore integrating the game mechanics into the curriculum. When programming a game, it is important to stick to game criteria [5] and to understand the structure of games. One of the most popular tools for creating games is the Mechanics Dynamics Aesthetics (MDA) framework [6]. This game design process has the following structure: Developing the core idea, writing a game concept, producing the artwork, programming the game engine, producing the game content, testing, balancing, and fixing the bugs. Typical game elements include players and their roles, objectives, procedures, rules and underlying game mechanics, resources, conflicts, obstacles, opponents, and an outcome. Teachers should consider how to support collaboration and communication during the whole game creation process. The teacher also needs to foster teamwork and consider the different preferences of students, e.g., if they feel more confident in the role of developers or artists. Finally, the teacher should ensure that the ideas for games are simple and clear, as well as reduce the size and complexity of the game projects.

Games, in contrast to playing, are characterized by explicit rule systems towards discrete goals or outcomes. Therefore, they are an innovative learning approach and possess educational value [7]. Engaging activities are in general, well received by students and contribute to improved overall motivation and productivity. Within a game-based learning environment, students can unleash their own creativity, express themselves, and connect with their classmates, all of which promotes their social inclusion [8]. Learning is an active process based on the learners’ interests, curiosity, and experience. Establishing such an educational practice requires an understanding of new learning principles and content-based curriculum issues, students’ collaborative learning processes, and the development of new concepts of projects based on learning knowledge. In groups, students can solve challenging problems that are open-ended, curriculum-based, and often interdisciplinary [9].

The intention of using games within education involving programing is to motivate students; especially in the Science, Technology, Engineering, and Mathematics (STEM) classes. Programming is not a single skill but more a complex activity, where a student must apply cognitive skills (such as abstraction) to solve a single task [10]. A lack of motivation and a missing sense of achievement can lead to negative programming experiences. Li and Watson [11] divided game-based learning computer courses into three main categories: 1) The authoring-based approaches, which refer to game themed assignments [12]; constructing, completing, or modifying existing games; the use of graphical and simplified learning tools [13,14]; or providing frameworks. The visual programming environments allow novice users to keep the focus on the semantics of programming and problem-solving aspects and eliminate the need to deal with syntactical problems. 2) The play-based approach, which allows learning to program through game playing. The students can develop code while playing tutorial games [15], debug a game [16], or use multi-player real time strategy games [17]. 3) The visualization-based approaches: use micro-worlds and demonstrates code execution in a visual context [18].

Using a gamification approach [19], attempts to keep users motivated to perform certain tasks [20,21,22] via integration of elements, such as adding points, badges, and leader boards [23,24]. Gamification involves using game-based mechanics, aesthetics, and dynamics to engage people, motivate them, promote learning, and help them in problem solving [25]. The goal is to improve the user experience (UX) and user engagement, which requires a deep understanding of the users’ needs. In such settings, the players perform tasks and are rewarded for them, which should increase their engagement. However, not reaching their learning goals can lead to stressful situations, which discourages the users from continuing to play [26].

### 1.2. The Approach and Motivation

The capabilities of modern mobile devices, smartphones, and tablets, as well as data acquired by the built-in sensors in these devices (e.g., accelerometer, gyroscope, magnetic sensor, or light detector) support the creation and coding of applications that enable the simulation or re-creation of real experiments for subjects, such as physics or math [27]. By connecting with sensory devices, the dynamic information about the environment (e.g., light or temperature) is tracked. Also, patterns of the object’s movements (e.g., the use of accelerometers handling axis-based motion sensing data together with gyroscope data for mobile orientation to enable racing game templates or developments) can be understood. Moreover, using sensors in mobile devices enables not only their use as sensing nodes, but also affords other opportunities via their embedded practical tools that enhance the UX, including touch technology, global positioning system (GPS) navigation, vibration modes for sensing [28], multiparameter sensing, link with other games or robots (e.g., Lego), and playing of games by moving the phone in different directions [29].

**Figure 1:**
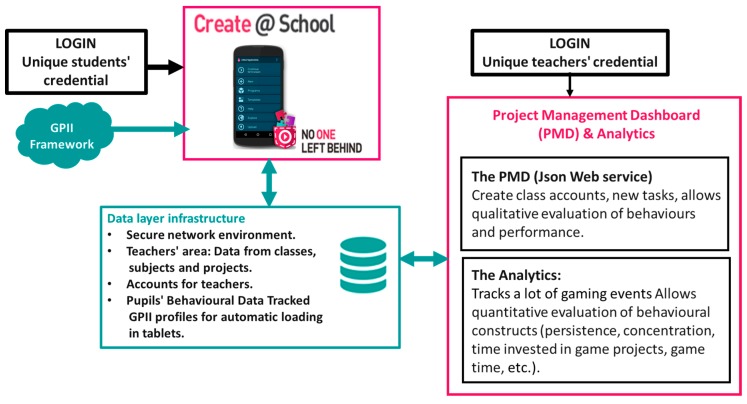
High level view of the No One Left Behind (NOLB) technical framework.

The No One Left Behind (NOLB) project [30] brings together the concepts of constructivism, games, game-based learning, and gamification into an integrated technical framework that comprises a set of digital tools. The main technical framework encapsulating these tools is shown in Figure 1. The framework comprises the following complementary technologies, in order to enable game-making development and inclusive participation:

- The Create@School App, targeting the students; and
- The Project Management Dashboard (PMD) [31], targeting their educators.

The Create@School App is based on the Pocket Code Framework, which allows the creation, playing, sharing of remix programs using a “Lego- brick style" [32]. Children can code games, animations, interactive music videos, and many other types of apps, directly on the phone or tablet. Pocket Code was inspired by Massachusetts Institute of Technology (MIT)’s Scratch project [33]. With Scratch, teens can also program and share their own interactive stories, games, and animations in classes and the online community. Other similar applications, such as Minecraft [34], TinyTap - Educational Games [35], GameSald [36], Bloxels [37], and Gamestar Mechanics [38] are considered good resources to support the creation of game-making apps, games, and websites; balancing the fun of play with the challenge of coding and design academic content in classes. However, these apps enable and the development of programming coding skills in children, but have not been designed as customized applications for code-making in academic environments.

The Create@School App is the entrance point to the system for students and is deployed in mobile devices. The Create@School App takes advantage of mobile devices’ hardware capabilities, utilizing the possibility of using various sensors and expanding the sensoring capabilities. The Global Public Inclusive Infrastructure (GPII) allows automatic settings to be implemented on the mobile devices, allowing accessibility for students with special educational needs (e.g., bigger sizes of text for easier reading, appropriate colors and contrast for those with visual impairments, vibrations, etc.). A data layer infrastructure is linked to a web-based environment, providing the data repository, network, and security infrastructure.

The Project Management Dashboard (PMD) is the entrance point for teachers, allowing their interaction with the collected data (i.e., game-based projects submitted by students in classes). This infrastructure stores and provides access to the teachers´ classes, allowing the maintenance of data from projects´ evaluations, games revision from students, and students´ monitoring as well as evaluation of behavioral data. The PMD comprises the analytics module, which analyzes the students’ movements and the behaviors using big data. This provides tangible measurements of socio-behavioral constructs (e.g., persistence, sharing, time consumed, analyses of blocks used, etc.).

This paper describes the context, in which the Create@School App (including the pre-designed stage called Pocket Code) and the PMD were validated and tested, as well as the assessment approach used to evaluate the UX. The UX evaluation comprised attributes or measures, such as usability, utility, and attractiveness of these developed technologies. These attributes were evaluated by students and teachers as products for education. Relevant findings of the evaluations performed in the NOLB pilot deployed in Spain are discussed, and future improvements in order to bring the tools to the market are proposed.

## 2. Materials and Methods

The solution presented in this manuscript provides the design, development, and evaluation of the Create@School App and PMD. This solution aims at developing content rich applications as a result of digital game-making and self-evaluation of created artefacts, to help students and teachers to better explain their academic content, especially in subjects, such as math or sciences. To accomplish this aim, the following materials and methods were applied.

### 2.1. Materials

The Create@School App collects data from students’ surrounding environment through the mobile devices they interact with, and interacts with the PMD; for this purpose; the following materials were used:

- Pocket Code Framework [32]: An open-source framework for mobile devices that allows children to create their own games, animations, music, videos, and many types of apps, directly on their phones or tablets. Pocket Code provides pre-coded modules, so called bricks, that enable connection with mobile devices sensors, as well as links with other sensor-based approaches and developments, such as LEGO Mindstorms®. Pocket Code is the bais of the Create@School App and was used as a pre-designed version of this App to train both students and teachers, in coding skills.
- The Create@School App is an integrated development environment (IDE) for smartphones and tablets designed for children. It is the enhanced version of Pocket Code that has been customized for educational environments. The Create@School App embeds the concepts of game mechanics and dynamics through ready-to-use (pre-coded) game templates (based on different game genres coming from a leisure gaming environment). Students used the Create@School App in class, to integrate playful activities into regular classroom education. This kind of classroom setting allowed a hands-on approach to provide extrinsic motivation for students when starting to use a new tool [39]. Students need some time to start benefiting from new educational tools, and thus may take longer to become intrinsically motivated.
- Templates: Pre-coded templates were developed integrating nested objects as object collections (i.e., grouping several objects), clustered levels through scenes, or pre-coded interaction with a sensor. These templates were prepared (coded) in Pocket Code and linked to the different academic competences for different subjects and classroom ability levels. The templates allowed teachers and students to develop any kind of game genres in a standardized manner; being flexible enough to adapt to the preferences and likes of the students, and affording different methods to present and play with the academic content.
- The Global Public Inclusive Infrastructure (GPII) framework is an infrastructure that allows accessibility preferences to be set (e.g., text with bigger font sizes, appropriate colors, etc.) for children and people with special educational needs, making the Create@School App a more accessible IDE.
- Project Management Dashboard (PMD) and analytical tool: A web interface that allows orchestration of the class environment and enables the integration of information from all students in class, including the list of students per class, the projects assigned, and evaluation of the projects about the academic or curricular objectives. Through the PMD, the teachers not only can plan, assign, and manage the delivery of game projects to support new game-based teaching approaches, but can also evaluate students regarding the completion of projects and achievements of academic objectives. The PMD is based on the idea of implementing a game jam approach in classes [40]. This enables collaboration, engagement, and competition between the students that develop each project. The created game projects are uploaded to the PMD by each student at the end of the lesson.
- The analytical tool is embedded into the PMD and allows monitoring and assessing of the way the students’ work and code on a class project, thus providing a set of quantitative values that enables the evaluation of socio-behavioral constructs; including: confidence, self-efficacy, performance, interest, creativity, persistence, effort/dedicated time, and concentration amongst others. Thus, the analytical tool generates feedback data on each student’s progress, socio-behavioral constructs, coding, and use of elements of the Create@School App, streamlining the process of the assessment of student work.
- Tablets and mobile phones: In total, 338 tablets and mobiles devices were used for the classes comprising the following models and specifications: Seven-inch and 10-inch Android mobile devices, which include Google Nexus 7, MOTOG-2 as well as BQ Edison 3 10-inch and 8-inch. The resolution was: either 800 × 500; 1024 × 640; or 1280 × 800—a common aspect ratio of 1.6. The devices had a range of embedded physical and virtual sensors. The physical sensors were hardware-based sensors embedded directly into mobile devices that derive their data directly by measuring particular environmental characteristics (e.g., accelerometer, gyroscope, and proximity, etc.) and virtual sensors that were software-based, harvesting their data from several hardware-based sensors (e.g., in the Android platform - linear acceleration, and gravity sensors).
- LEGO MINDSTORMS® technology [41]: A programmable robotics construction set that allows the building, programming, and commanding of LEGO robots from their PC, Mac, tablet, or smartphone. It provides an interface to enable programmable intelligent bricks or modules, thus it is able to interact with Pocket Code. It comes as a set that includes connecter and universal serial bus (USB) cables, LEGO Technic pieces or elements, one EV3 Brick, two Large Interactive Servo Motors, one Medium Interactive Servo Motor, and touch, color, infrared, and infrared beacon sensors.

### 2.2. Methods

#### 2.2.1. The Sample

In the No One Left Behind project, a two-cycle experiment was planned for three pilot sites, which were in Spain, the United Kingdom and Austria, reaching over 600 students from eight different schools. Due to the different contexts of the three pilot sites, three different evaluation studies were planned within the project. In Spain, the pilot focused on the validation and evaluation of the developed tools as products for education.

This paper presents the results of the Spanish pilot evaluation. This was performed in two schools located in two different Andalusian cities (Úbeda and Puerto de Santa Maria). A total of 308 students participated in the pilot and technically validated the tools during the two cycles, while 115 of them valorized the user experience. The students’ age ranged from 8 to 17 years, and those studying courses from 6th to 11th grades participated in the evaluation. Of those, 45% were girls and 54% were boys.

In addition, 16 educators participated in the evaluation of both the Create@School App (6 of them used the preliminary version in Pocket Code, 6 used the Create@School App, and 4 used both versions) and the PMD. 

The pilots adhered to the national guidelines in terms of ethical considerations and principles, using and providing amongst others: (a) Parental informed consent; (b) provision of information specifying the alternatives, minimal risks (i.e., not to be exposed to minimal risks), participation conditions and benefits for those involved before consent was sought; (c) anonymity; (d) confidentiality; and (e) in-detail information to stakeholders (e.g., the European Commission - EC) regarding ethical aspects of research and evaluation/validation in reporting activities.

#### 2.2.2. The Technology Validation Cycles

The project and all the pilots were set for two consecutive academic years, each year corresponding to a project cycle.

During the first cycle (i.e., first year of the project), the Pocket Code mobile application [42] was used in classes, as it can be considered a pre-design stage version of Create @School. Also, during this first cycle, an initial set of templates and technical modules were generated to enable an easy to use game context and game elements within the academic curriculum topics. The curriculum of the subjects comprised: Science, enrichment (i.e., a multidisciplinary program available in Andalucía), mathematics, and the program for children with attention deficit disorders (PEMAR; a special program for children with attention deficit disorders). The involvement of students and teachers participating in each of the pilots by school year, age, and subject is depicted in Table 1.

During the second cycle, the Create@School App and the PMD were completed, refined, and validated, involving pupils and teachers as presented in Table 2. During this cycle, the technologies were deployed to be validated in the following academic subjects: Computing, mathematics, science, programming basics, biology, geology, language, social sciences, and enrichment.

Table 2 shows the scenario of the second cycle (i.e., the second consecutive full academic year). During that year, the teachers and students used, validated and iteratively improved versions of the Create@School App and PMD applications.

#### 2.2.3. Template-Based Methodological Approach

NOLB used game-based templates, which were generated during the project in order to standardize the initial coding stage, to manage the time for each class in an efficient manner. The templates also integrated gamification elements and pre-coded modules that supported the capturing of the academic content and processing of data generated from sensors. The templates could be customized to academic subjects and personalized towards users´ preferences. The goal of these templates was to give students an opportunity to start with an almost finished game [43] (game mechanics pre-coded) and apply game-making to any kind of curriculum subject.

#### 2.2.4. Measures and Evaluation

The validation of the Create@School App was performed by students and teachers, while the PMD was validated by teachers alone. The PMD tool was developed within the project in order to have an integrated environment where educators could review and evaluate the games and apps developed by the students for each class context. The Create@School App integrated the results from the teachers´ observations, which were used to better inform the use of the Create@School App in educational environments.

##### Hassenzahl Model and AttrakDiff Tool

The Hassenzahl model [44] is designed for measuring a user’s experience and satisfaction and for understanding the user’s emotional responses, thus providing information on the acceptability and usability of the developed apps.

The Hassenzahl model was selected for use in the project as it provides an a priori defined method for pre-post design of the apps. It also allows the comparison between the two applications. The comparative capacity of this methodology fitted well with the fact that for the first cycle of validation, the Pocket Code App was used in classes (as a pre-design version of the Create@School to train both students and teachers with coding skills), while during the second cycle, the Create@School App (i.e., enhanced version of Pocket Code) was utilized. Thus, the Hassenzahl model with standardized *AttrakDiff* surveys allowed the comparison of the Pocket Code and Create@School applications, as well as providing the UX insights from the above-mentioned users.

The Hassenzahl model uses the AttrakDiff surveys [45] as a tool for gathering responses regarding the qualities of physical products, websites, software, and other digital media, and thus, modelling user experience and satisfaction, as well as understanding emotional responses. In this approach the following issues are considered:

- The usability and utility of the technologies perceived by the users;
- The satisfaction of the users that used the technologies, and the attractiveness of the technologies.

Based on the Hassenzahl model, the qualities of physical products, websites, software, and other digital media can be classified into two distinct groups:

- Pragmatic qualities (PQs): These attributes are related to practicality and functionality. A consequence of pragmatic qualities is usefulness and usability. The pragmatic quality (PQ) scale has seven items, each with bipolar anchors that measure the pragmatic qualities of the product. This includes anchors (see) such as technical–human, complicated–simple, confusing–clear, and impractical–practical, among others.
- The hedonic qualities (HQs) reflect the psychological needs and emotional experience of the user. In the Hassenzahl model, hedonic qualities are divided into two categories:
  ○ The stimulation quality (HQ-S) represents the users wants to be stimulated in order to enjoy their experience with a piece of software or product. These include rarely used functions that can stimulate the user and satisfy the human urge for personal development and increased skills. The hedonic stimulation quality (HQS) scales have seven anchors each. HQS has anchors, like typical–original, cautious–courageous, and easy–challenging.
  ○ The identity quality (HQ-I) refers to the human need of expressing through objects, to control how people want to be perceived by others. Humans have a desire to communicate their identity to others through the things they own and the things they use. They help humans to express themselves; who they are, what they care about, and who they aspire to be. The hedonic stimulation quality (HQS) scales also have seven anchors each. HQI has anchors, like isolating–integrating, gaudy–classy, and cheap–valuable.
  ○ There are seven items for overall appeal or attraction (ATT), which comprises opposite words scales (e.g., ugly–beautiful and bad–good). The items are presented on opposite sides of a seven-point Likert scale, ranging from −3 to 3, where zero represents the neutral value between the two items of the scale.

The Hassenzahl model was used to validate the pragmatic and hedonic perception of Create@School App (by teachers and students) and PMD (by the teachers). Overall, the AttrakDiff measures provided the pragmatic (practicality and functionality) and hedonic perception (emotional experience), which in turn enabled the measures to be used in the evaluation, which comprised usability and acceptability (i.e., user experience, satisfaction, and emotional responses).

##### Competitive Validation Outside Classes Environment

The pilot school in Ubeda used the Pocket Code and Create@School Apps for the LEGO® League [46]. The LEGO® League Open European Championship is an international robotics tournament hosting more than 90 teams from more than 80 countries all over the world. It presents an international platform that inspires future leaders and innovators in science and technology through challenges, competitions, and teamwork. Young candidates aged 10 to 16 years old are guided by adult coaches to research a real-world problem related to their interests, such as food safety, recycling, energy, etc. Then, students are challenged to come up with a solution. This process requires the students to design, build, and program a robot with LEGO MINDSTORMS® technology [47]. The competition takes place on a table-top playing field. There is a programmable robotics construction set that gives the power to build, program, and command LEGO robots. The LEGO MINDSTORMS® technology includes everything needed to create robots and make them walk, talk, move, etc., including touch, color, and infrared beacon sensors. LEGO provides free software and connects with the Pocket Code App, allowing children to build, program, and control the robot from tablets or smartphones.

At the pilot school, there is a program for high capacity students that includes extracurricular activities, including robotics using LEGO NTX and EV3 robots. The Pocket Code and Create@School Apps were used for prototyping a solution linking and exploiting the data acquired from the LEGO® robots on tablets and mobile phones´ sensors.

## 3. Results

### 3.1. Design and Development of Create@School App and PMD

The main results of the project comprised the development of the interactive Create@School App and PMD, which were evaluated to analyze their acceptability and satisfaction. The Create@School App was co-designed and co-developed with students and teachers during the first cycle of the project, using Pocket Code as a pre-design version of the Create@School App. During this cycle, the game-design was integrated, allowing students and teachers to be trained and familiarized with the coding language, developing coding skills, gaining knowledgeable on the mobile sensors and capabilities for coding, as well as creating the procedures to integrate coding as part of the classes and as a tool for classroom projects. The Create@School App had the following improvements over its pre-designed version (Pocket Code):

- Customization for education environments;
- Integration of game templates, providing pre-coded templates that have coded game mechanics (i.e., templates for adventure, action, quiz, or puzzle games). This reduces the time needed to develop games and applications in classes, as well as allowing personalization for different ages, personal interests, and academic content through the development of the game dynamics and aesthetics by pupils. The templates and the Create@School App enable game dynamics and aesthetics by the editing of an existing game design, whilst allowing personalization of backgrounds, landscapes, and characters, the creation of new challenging levels, as well as changing the difficulty of a game;
- Integration of 48 new features and improvements identified during the project and based on the experience and insights gathered from the No One Left Behind pilots’ users;
- Integration of the GPII framework that allowed automatic and individual personalization for students with learning disabilities and additional sensory impairments.

The PMD web area was co-created with the schools´ teachers. The feedback was received from end users regarding requirements and needs for interface designs, functionalities, and workflow. The data production and consumption flow were supported by the interaction of the Create@School App with the PMD processing backend, enabling a data ecosystem which supports teachers to track the coding and academic progress of their pupils, while using the application.

### 3.2. Evaluation Results

The following sections present the description of the results gathered from the Create@School App and PMD user experience evaluation.

#### 3.2.1. Results of the Evaluation of Pocket Code vs. Create@School by Teachers

The AttrakDiff survey was used to gather the information from the teachers that evaluated both applications: Pocket Code and Create@School. Results were integrated into the Hassenzahl model in order to provide tangible values to the attributes and measures evaluated.

**Figure 2:**
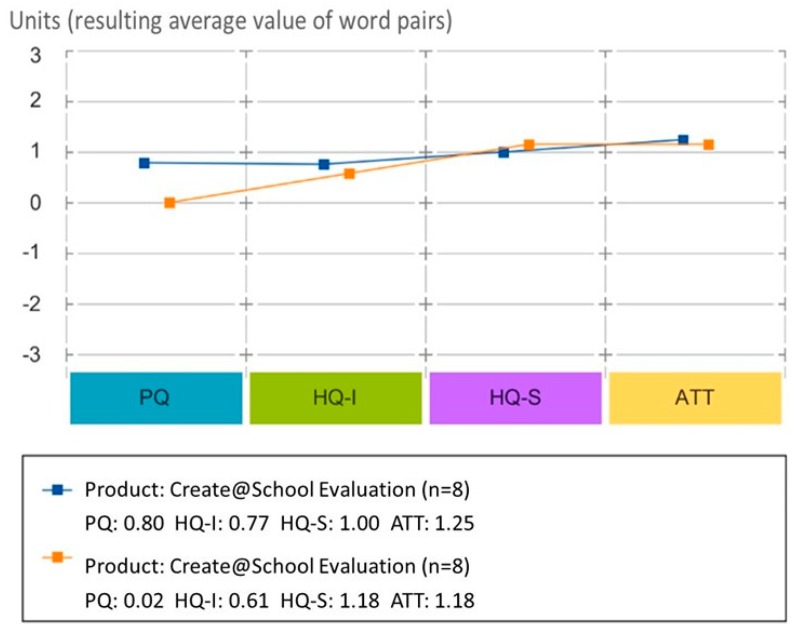
Diagram of the average values for pragmatic qualities (PQs), hedonic qualities: hedonic identity quality (HQ-I), hedonic stimulation quality (HQ-S), and attractiveness (ATT) for the study 3.2.1.

Figure 2 presents the average values for the evaluation of each apps’ attributes. It can be observed that both products (Pocket Code vs. Create@School App) have a good balance between pragmatic and hedonic attributes. However, the Create@School App scored higher in pragmatic and hedonic attributes. Pragmatically, the Create@School App was perceived as more task-oriented and easier to manipulate when aiming at fulfilling the goals (teaching/learning goals), while hedonically, the Create@School App was perceived as more self-oriented and easy to identify within the social context, as well as for the development of the skills. Both, the Pocket Code and. Create@School Apps were positively accepted by teachers, but at this stage, these were still not considered as the desired products.

Users had a wider spectrum of differing opinions regarding the Create@School App (see Figure 2) compared with the Pocket Code App. The largest positive distance between the Create@School and Pocket Code Apps is seen in pragmatic quality, as Pocket Code scored the lowest in this attribute (the pragmatic quality mean is 1 point lower than Create@School, as well as any other mean in the other measured attributes for Pocket Code). This indicates that this coding application has fewer pragmatic qualities than identity and hedonic ones. For identity (HQ-I), the Create@School App is slightly better than Pocket Code, while for stimulation (HQ-S), Pocket Code is slightly better than the Create@School App. Neither of the products reached the maximum rating in any quality.

**Figure 3:**
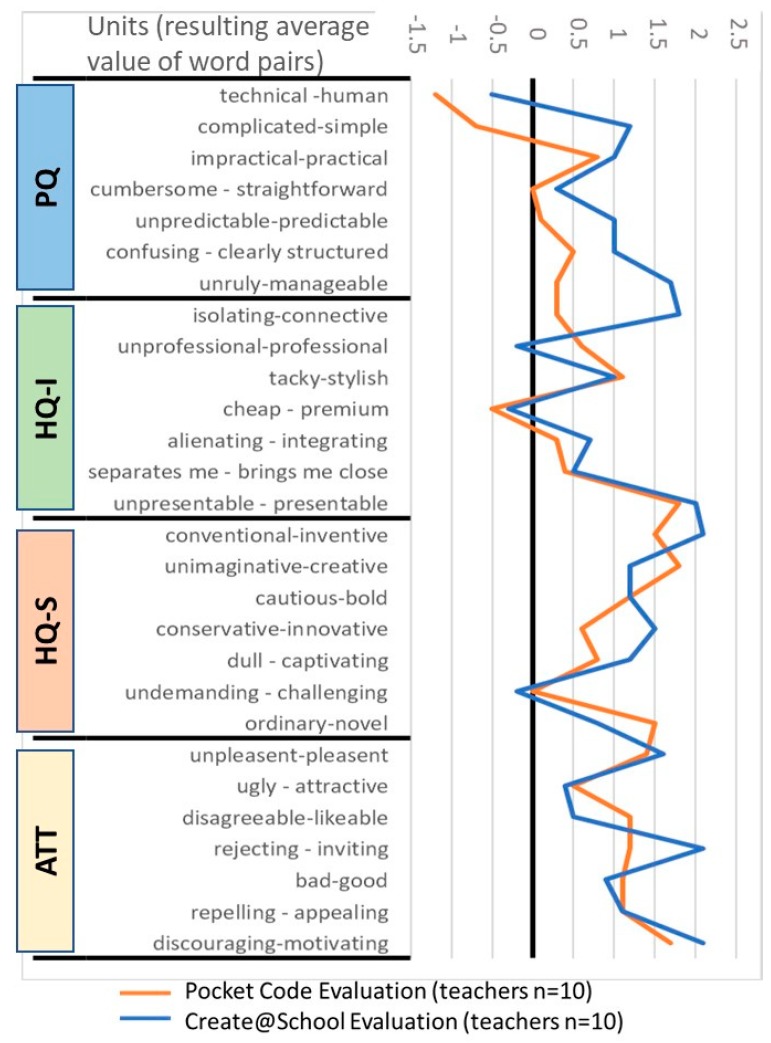
Diagram of the average of word pairs averages grouped by pragmatic qualities (PQs), hedonic identity quality (HQ-I), hedonic stimulation quality (HQ-S), and attractiveness (ATT) for the study 3.2.1.

The diagram in Figure 3 shows the results from the semantic differential of the model and presents the average results of the word pairs (opposing attributes presented as a seven-point Likert scale, ranging from ‒3 to 3 (adjusted in Figure 3 to ‒1,5 to 2,5 for better visualization of results), where zero represents the neutral value between the two items of the scale) from each group: Pragmatic (PQ), identity (HQ-I), stimulation (HQ-S), and attractiveness (ATT) for the Pocket Code (blue line) and Create@School (orange line) apps.

In this diagram, the Create@School App scores better than Pocket Code in characteristics, such as simplicity, manageability, and providing a clear structure in PQ, alienating or integrating with interests in HQ-1, inventive and captivating in HQ-S, as well as inviting and motivating in ATT. Pocket Code scored better than Create@School in professional look, innovativeness, and novelty.

#### 3.2.2. Results of the Evaluation Study of Project Management Dashboard (PMD)

This study collected the evaluations of all the teachers that using the Project Management Dashboard (PMD) in the Spanish pilot. The surveys were collected at the end of the second academic year.

**Figure 4:**
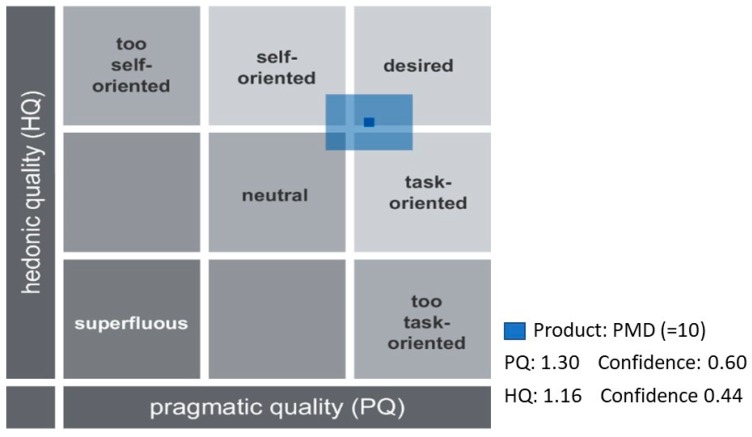
Overall evaluation of hedonic and pragmatic qualities for the study 3.2.2.

The combination of HQ and PQ provides the evaluation of the PMD interface positioned in a matrix that according to the model has pre-established some values that characterize the HQ and PQ combination (e.g., self-oriented, neutral, task oriented, etc.; see Figure 4). The confidence rectangle (smaller means higher confidence and larger means less confidence) shows that the hedonic quality is close to the pragmatic quality, characterizing mostly the interface as practical. For PMD, the confidence rectangle extends from the desired area and into the self-oriented and desired areas. Therefore, this means that users provided different ratings, tending to provide high scores for these attributes (desired, task oriented).

**Figure 5:**
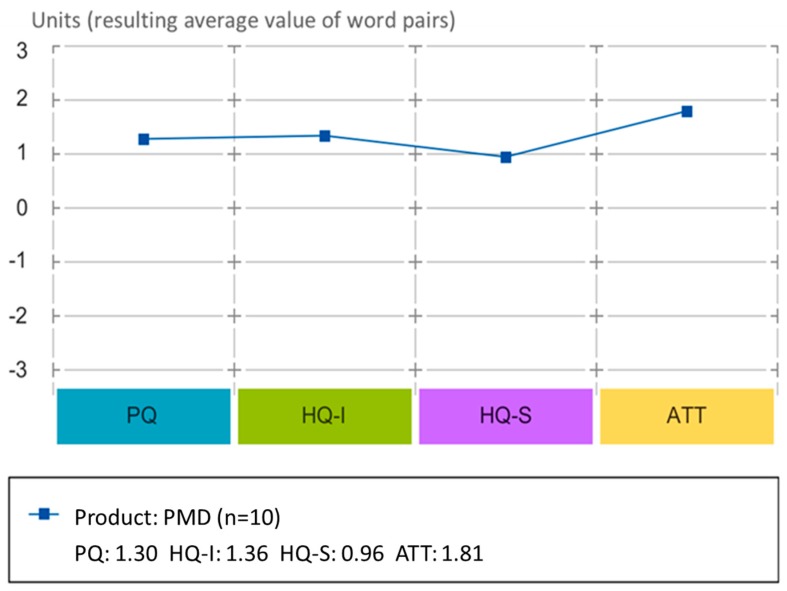
Diagram of the average values for pragmatic qualities (PQs), hedonic identity quality (HQ-I), hedonic stimulation quality (HQ-S), and attractiveness (ATT) for the study 3.2.2.

The average of values of the AttrakDiff dimension of the PMD (Figure 5) shows the different values of stimulation, identification, and attractiveness. In this regard, all of the PMD attributes received positive evaluation and maintained themselves above the average region (between 0 and 2, not much outstanding attributes) except for attractiveness, which has a slightly higher evaluation than the rest of the attributes.

**Figure 6:**
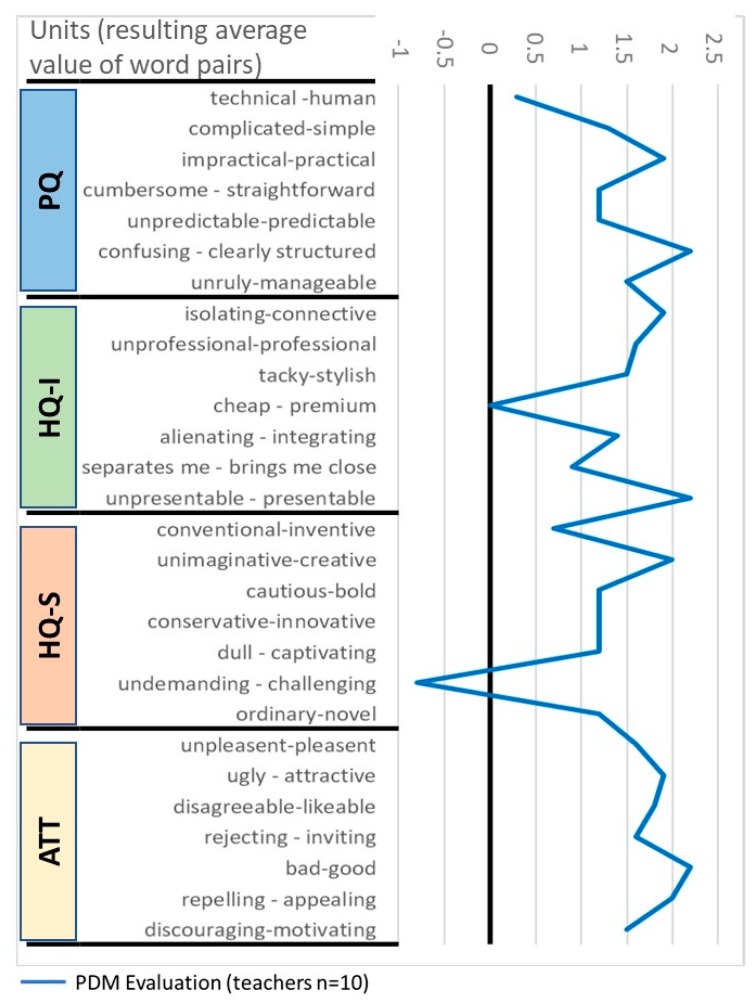
Diagram of the average of word pairs averages grouped by pragmatic qualities (PQs), hedonic identity quality (HQ-I), hedonic stimulation quality (HQ-S), and attractiveness (ATT) for the study 3.2.2.

Figure 6 shows the details of the semantic differential of the surveys. When going through the specific attributes (given by the word pairs) in each quality segment (PQ, HQ-I, HQ-S, and ATT) for the PMD, it can be observed that the solution goes out of the average values, characterized by having a clear structure in PQ, being highly presentable and connective in HQ-I, creative in HQ-S as well as attractive and appealing in ATT. Its technical character, affordability and undemanding interface were appreciated.

#### 3.2.3. Results of the Evaluation of Create@School App by Students

The validation process collected the evaluations of students that used the Create@School App in the Spanish pilot during the second cycle of the experiment. In total, 115 students participated in the evaluation of the Create@School App in the different subjects.

**Figure 7:**
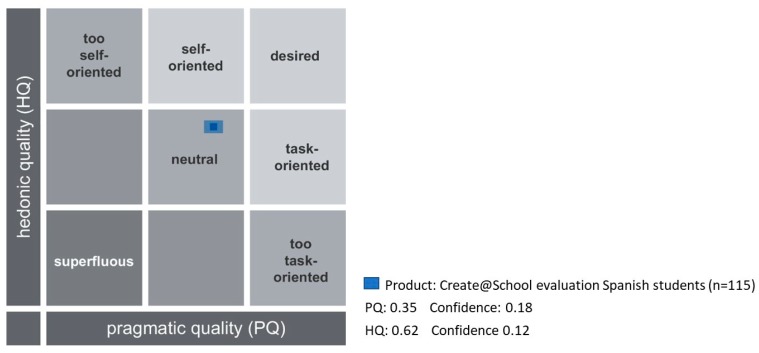
Overall evaluation of hedonic and pragmatic qualities for the study 3.2.3.

The confidence rectangle (Figure 7) is very small, which shows a high coincidence among answers. According to the average answers from the students, the Create@School App was rated as neutral in terms of pragmatic and hedonic attributes.

**Figure 8:**
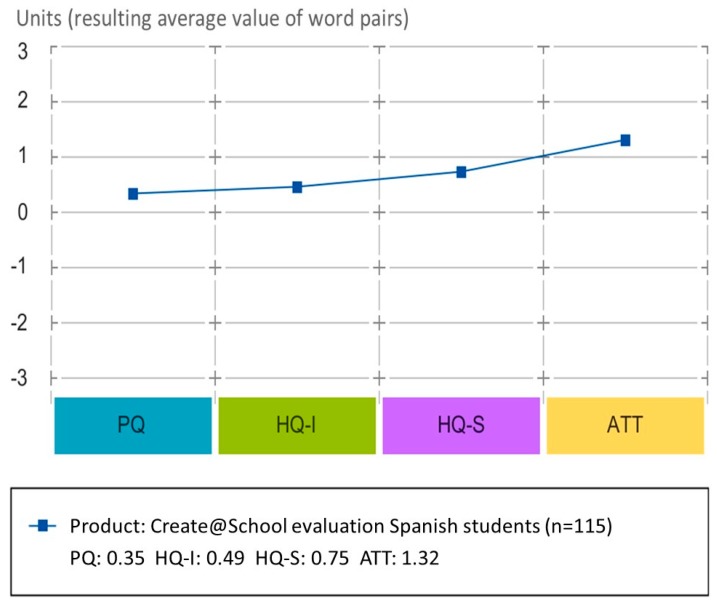
Diagram of the average values for pragmatic qualities (PQs), hedonic identity quality (HQ-I), hedonic stimulation quality (HQ-S), and attractiveness (ATT) for the study 3.2.3.

Similarly to the PMD, the average values of the AttrakDiff dimension of the evaluated App (Figure 8) by students shows the different values of stimulation and identification are in the average region (between 0 and 2—not much outstanding attributes) while attractiveness has a slightly higher evaluation than the rest of the attributes.

**Figure 9:**
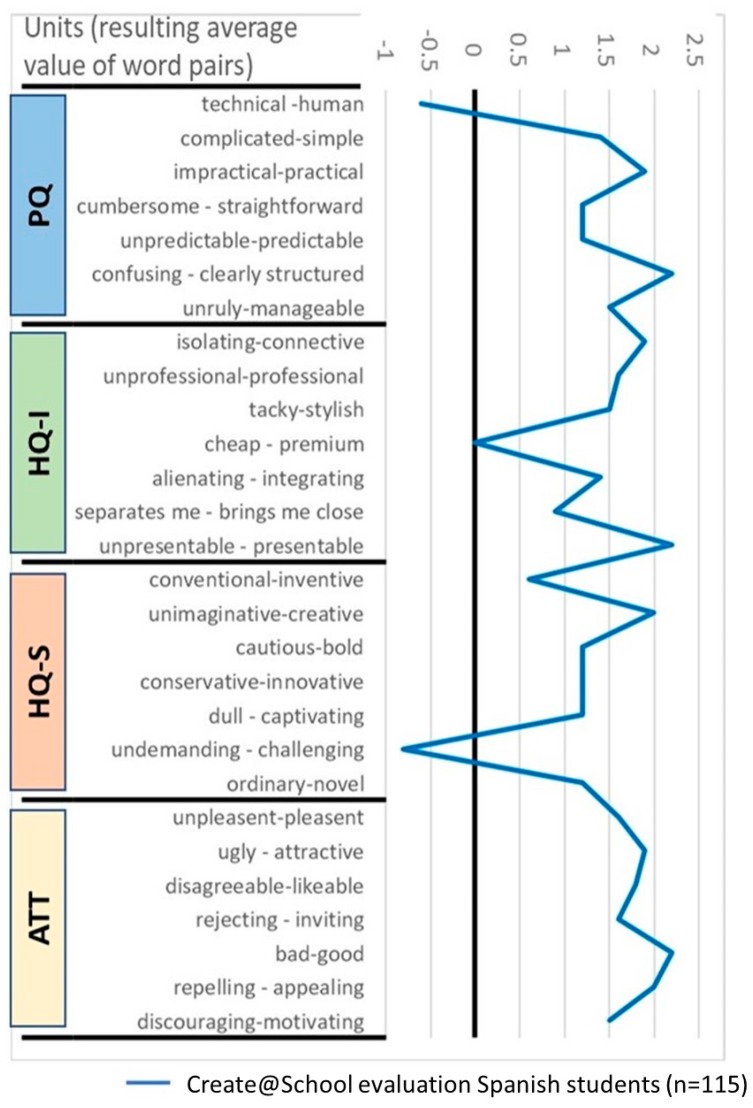
Diagram of the average of word pairs averages grouped by pragmatic qualities (PQs), hedonic identity quality (HQ-I), hedonic stimulation quality (HQ-S), and attractiveness (ATT) for the study 3.2.3 (adjusted scales to -1 to 2.5 for better visualization of results).

The diagram for the semantic differential of the surveys (Figure 9) shows that Create@School App is characterized as being perceived as a practical, connective, stylish, presentable, creative, easy to use (not challenging), and an appealing App, while being considered as affordable and technical-oriented.

#### 3.2.4. LEGO NXT, EV3, and LEGO® League Validation

Some of the students who participated in the project developed a robot that separated organic from non-organic waste, allowing them to control the robot with many advanced Pocket Code and Create@School blocks, using mobile sensors to direct the robot as well as integrating LEGO® sensors for the detection of obstacles while the wheeled robot moved forward (or backward). This project enabled identification of the most appropriate way for capturing and sharing the Create@School code, while encouraging personalization and customization of the developed code to the educational domain (e.g., using text, voice, images, colors, sensoring capabilities, such as opening doors for throwing waste away, and other features that relate to the natural science and/or waste treatment domain).

The Úbeda Team ranked second place in the local LEGO® League championship of Granada, leading them to participate in the provincial league. Here, they won the award for robot designers and the award for young promise.

## 4. Discussion

This research describes the evaluation of the Create@School and PMD tools, developed as part of the No One Left Behind project. Results of the evaluation of these tools provide insights regarding students´ and teachers´ perceptions after using them for a period of at least one year.

### 4.1. Teachers’ Evaluation of Pocket Code vs. Create@School Study

The Pocket Code and Create@School Apps achieved very similar scores in the evaluation, which was initially expected, as the Create@School App was built to be education-oriented and as a simplified version of the Pocket Code App to be used in the classroom context. Both, the Pocket Code and Create@School Apps were positively accepted by teachers and with a balanced perspective (pragmatic vs. hedonic). However, the apps were rated as neutral by students. This was driven by several factors: The applications were selected by the school, thus they were considered as a class resource; also, some external factors, such as poor Wi-Fi connection in classes and some authentication problems for security and privacy (preventing free internet navigation at school), caused frustration while using these apps. However, in this context, the Create@School App was perceived to be more task-oriented to the educational domain than Pocket Code; being perceived mor informal and more like a game app for entertainment.

The evaluation of pragmatic quality (PQ) is related to the practicality and functionality of a system and is also an indicator of its usefulness and usability. Figure 2 and Figure 3 show the difference between the Create@School and Pocket Code Apps. Figure 3 shows that the biggest difference between the two applications is appreciated in the pragmatic quality (PQ), which collects all attributes related to practicality and functionality. The Create@School App is perceived to be more usable and useful than Pocket Code for the educational domain. This was driven by the integration of game templates, which saved more than 40% of the coding time and allowed pupils to more easily integrate images and sensors.

Additionally, the extreme values seen in Figure 3 represent the most significative characteristics of the application. In this regard, the Create@School App is more manageable, connective, inventive, presentable, inviting, and motivating. The Pocket Code App is seen as more creative, innovative, and novel. The novelty and innovation of the Pocket Code App over the Create@School App was driven by the fact that Pocket Code is a precursor to the Create@School App, and thus the first impact when using it was higher the first time of use.

### 4.2. Teachers’ Evaluation of the PMD Study (3.2)

The PMD satisfied the teachers’ expectations and was rated as a desired product. The PMD was designed to support the evaluation of Create@School projects and provided the socio-behavioral measures that were not managed by teachers (such as persistence, concentration, sharing, etc.). The positive assessment of the PMD shows that providing tangible and quantifiable feedback to teachers regarding behavioral constructs was useful, usable, and stimulating, supporting the use of the Create@School App in classrooms for academic-oriented projects, as a new tool for education. However, the fact that the PMD was not fully integrated into the current school management system to exchange data (such as grades of the projects) diminished the attractiveness of the application.

### 4.3. The Students’ Evaluation of Create@School Study (3.3)

The Create@School App satisfied the pupils’ expectations even if it was not yet their desired product, and the application was still accepted. It was perceived as useful and usable, attractive and stimulating, being a good foundation product for the creation of a new innovative tool for education. It was rated as good, practical, and creative. This is driven by the sense of empowerment allowing students to produce their own content, as well as being able to build a game that could be played by with their peers and friends. Moreover, pupils liked the idea of having teachers and students working together with the applications. This was considered a domain where students could demonstrate to teachers their higher programming skills on mobile devices. However, as teachers worked more as coaches (the evaluation was based on academic subject not the coding or computing skills), this brought a balance and a new role to teachers in the classes that was appreciated by the students.

Although classrooms had internet access for the many technology devices used, there were several external factors that affected the proper deployment of the Create@School technology in classrooms. The fact of not having a proper communication infrastructure that grows with demand (i.e., inadequate internet connectivity that generated disconnections) was perceived as a problem of the Create@School App, and not as an external factor affecting the use of the Create@School App. This issue also affected the evaluation that characterized the app as unpredictable and unruly. Also, by not providing open internet navigation (due to schools’ network security protocols and directives) through browsers (either to navigate or get images) during class hours, the App was more conservatively appraised.

### 4.4. The LEGO® League Participation

This approach allowed the Create@School App to be validated in a bigger and more competitive playful environment outside the school walls. The code developed in the Create@School App was connected not only with sensors from mobile devices but with sensors located in real objects (in this case, sensors located in the LEGO® robots). Moreover, although validation took place in a playful environment, the developments were still linked with day to day issues (e.g., recycling) and academic content from classes (i.e., recycling processes in science).

It is important to highlight that the LEGO® League competition (the usage of LEGO robots with Create@School) brought together all the concepts of gamification, game-based learning, use and exploitation of embedded, virtual, and external sensors as tools for enriching coding and user experience, problem solving activities, and competitions, which the No One Left Behind project aimed to test. The LEGO® League demonstrated a high educational value. Thus, code-making applications, such as the Create@School App, can take educational activities outside of the classroom, and generate empowerment providing a sense of informality and motivation. Independently of the awards received or the position achieved in the competition, the students experimented together on how to create new solutions, solve problems, and find the most appropriate way for capturing and sharing their creations in an independent way. The pupils were the authors of their own games with important themes addressed–i.e. environmental content. Additionally, a shared learning process was adopted to overcome obstacles in a collaborative intelligent environment, where improved communication skills for the presentation of their scientific work were stimulated.

Notwithstanding, the results of the evaluation of the Create@School App and PMD tools in the Spanish pilot demonstrated that the Game Making Teaching Framework and the related technologies have been accepted by teachers and students in this context.

Some relevant aspects were not evident during the preparatory and first cycles of the pilot but appeared during the validation or second cycle. In this regard, it can be concluded that the information technology infrastructure of schools, interoperability with the school management system, and features, such as parental control, are important factors for the deployment of technologies, such as Create@School and PMD. The fact that the infrastructure did not support enough devices connected at one time (e.g., collapse of the network or slow connectivity) generated problems with the interaction, and thus lowered the expected usability experience. Also, the operating system of the devices used during the trials did not include some of the schools’ requirements, such as a parental control application and a set of customizable settings for a better management of the device account. The PMD was not interoperable with the school management system, thus grades could not be automatically transferred there.

These drawbacks were time-consuming, and if avoided, could have resulted in an improvement in the validations results, from the perspective of both tools. Moreover, the sense of novelty to which students and teachers were exposed at the beginning of the project using the Pocket Code App diminished during the second cycle when using the Create@School App. This app was used as part of the day to day classroom activities during the second cycle, where thinking and game-based content development exercise were intertwined with the coding practice. Thus, at the end of the second cycle, the teachers and pupils had experienced a continuous game-based design approaches, where game and story crafting scaffolded knowledge representation. Although it was a positive sign of acceptability that the Create@School App was accepted as part of the classroom practice at the end of the second cycle, this measure of success diminished the sense of novelty of the application.

## 5. Conclusions

Overall, the results from the two-cycle pilot showed that the principles of computer programming promoted logical thinking and stimulated the creativity of students, whilst acquiring knowledge about the curriculum subjects. The Create@School App represents a technical resource for students and teachers in classrooms to allow the programming and design of games that can effectively support the development and adaptation of learning material in academic subjects, such as science, mathematics, history, or language. This provided new engaging methods for use, by both teachers and students in their classrooms. Previous literature supports engagement in academic subjects through gaming, as well as cognitive and affective benefits provided by this kind of game-based multimedia environment [2,3,11,12,32,40,42]; but through the evaluation processes described in this research, it has been shown that coding with the Create@School App was accepted to be used in formal and informal academic settings. The Create@School App was perceived as good, creative, practical, pleasant, easy to use, and connective when enabling an experience-driven game design process, while supporting reflection on the everyday academic teaching-learning experience.

It can be concluded that user experience methods captured the needs and feedback from users, generating educational value in classes and schools. This balanced perspective (hedonic vs. practical) provided good acceptance of the Create@School App between pupils and teachers. During the second cycle, the Create@School App was used and integrated as part of the academic subject’s assignments (defined as projects). The defined templates saved coding time, as the mechanics of the games were pre-coded (thus, time was effectively used in classes). Moreover, value generation was demonstrated through participation in the Lego® League teamwork. Also, the creativity of the Create@School App is supported by the empowerment of self-oriented students to produce game-based and designed-based learning to high standards. The teachers prepared students with tools (games, template-based apps, and Lego® hardware) that allowed the building and representation of their knowledge through Create@School coding, moving beyond the schools’ and classroom´ settings. This integration of knowledge comprised the one gained within the classroom (such as recycling from science classes) and from their experiences in the outside world. The data coming from sensors, including those located in real-world objects (i.e., in this case, Lego® robots) generated a very high and positive user experience and sensing capabilities for the Create@School App.

Further efforts should be undertaken to support students in classes, such as the creation of new templates and adaptation of pre-coded bricks to enable the more effective use of students’ coding time.

## Figures and Tables

**Table 1:** First cycle data.

School Site	Course	Age	Teacher Technical Background	Subject	Students
**Puerto Santa María**	Y11	15–16	No	Mathematics	30
	Y10	14–15	No	PEMAR—Mathematics and Science	13
	Y9	13–14	Yes	Mathematics	30
**Úbeda**	Y10	14–15	No	Science Methods	12
	Y9	13–14	Yes	Enrichment	12
	Y7	11–12	No	Science	25
	Y6	10–11	No	Science	24

**Table 2:** Second cycle data.

School Site	Course	Age	Teacher Technical Background	Subject	Students
**Puerto Santa María**	Y11	15–16	Yes	Computing	17
	Y10	14–15	No	Mathematics	20
	Y9	13–14	No	Science	9
	Y8	12–13	No	Mathematics	11
**Úbeda**	Y11	15–16	No	Programming basics	30
	Y10	14–15	No	Programming basics	16
	Y9	12–13	No	Mathematics, Biology, and Geology	21
	Y8	12–13	Yes	Enrichment	12
	Y7	11–12	No	Language, Mathematics, and Social Sciences	26
